# Prognostic value of C-reactive protein-albumin-lymphocyte index (CALLY) in patients with endometrial cancer under radical surgery

**DOI:** 10.3389/fcell.2026.1762287

**Published:** 2026-07-07

**Authors:** Yi Zhang, Lin Xu, He Li

**Affiliations:** 1 Department of Obstetrics and Gynecology, The First Affiliated Hospital of Soochow University, Suzhou, China; 2 Department of General Surgery, The Fourth Affiliated Hospital of Soochow University, Suzhou, China

**Keywords:** CALLY, endometrial cancer, NLR, nomogram, PLR

## Abstract

**Background:**

The C-reactive protein-albumin-lymphocyte (CALLY) index has been reported to possess prognostic utility in several malignancies. However, its relevance in endometrial cancer (EC) has not been fully elucidated. This investigation was designed to evaluate the prognostic predictive value of the preoperative CALLY index in individuals with EC undergoing radical surgery.

**Methods:**

A retrospective analysis included 288 patients from the First Affiliated Hospital of Soochow University as the training cohort, with 153 patients from Changzhou People’s Hospital serving as the validation cohort. The cutoff value and prognostic significance of the CALLY index were established using statistical analyses, and a nomogram was subsequently developed.

**Results:**

A decreased CALLY index was identified as an independent risk factor for overall survival among EC patients. The time-dependent AUC of the CALLY index for 1-year, 3-year and 5-year survival was numerically greater than that of conventional inflammatory markers, though the difference was not statistically significant. The nomogram integrating CALLY, age, International Federation of Gynecology and Obstetrics (FIGO) stage, and pathological subtype demonstrated strong predictive capability in both the training cohort and validation cohort, outperforming FIGO staging alone.

**Conclusion:**

The CALLY index is readily available, cost-effective, and may serve as a promising biomarker for preoperative prognostic evaluation in EC. The developed nomogram may facilitate individualized therapeutic planning and offer practical guidance for the precision diagnosis and treatment of EC.

## Introduction

Endometrial cancer (EC), representing one of the frequently occurring malignant tumors in women, typically arises from endometrial tissue and constitutes the sixth most prevalent cancer among females (Bray et al., 2024). In developed countries, the incidence of EC has shown a continuous upward trend owing to lifestyle alterations, population aging, shifts in dietary patterns, and other contributing factors, leading to EC being the most commonly diagnosed malignancy in women (Crosbie et al., 2022). According to data published by the International Agency for Research on Cancer of the World Health Organization, 420,000 new cases and 97,000 deaths were reported worldwide in 2022, ranking EC as the second most threatening reproductive system tumor to women’s health after cervical cancer (Bray et al., 2024). Although the currently established molecular classification system (e.g., polymerase epsilon [POLE] mutation, mismatch repair deficiency/microsatellite instability-high [dMMR/MSI-H], p53 abnormality, and no specific molecular profile) has provided critical support for clinical decision-making, it remains insufficient to fully elucidate the heterogeneous progression of EC, especially in poorly differentiated or non-endometrioid carcinoma subtypes, in which traditional tumor markers (e.g., cancer antigen 125 [CA125] and human epididymis protein 4) exhibit significant limitations in sensitivity and specificity. Therefore, a reliable, effective, and readily obtainable biomarker is needed in clinical practice to evaluate the prognostic risk of EC patients and guide treatment.

The association between inflammation and tumor development was first proposed by Rudolf Virchow in 1863. Inflammatory cells and cytokines may facilitate tumor growth and progression, and the severity of tumors may correlate with levels of inflammatory cytokines (Balkwill and Mantovani, 2001). C-reactive protein (CRP), functioning as an acute-phase reactant, demonstrates markedly elevated levels under inflammatory conditions and is regarded as a reliable indicator of systemic inflammatory status (Mahmoud and Rivera, 2002). Lymphocytes can exert a strong antitumor immune response and are linked to increased chemotherapy sensitivity and improved prognosis in cancer patients (Mahmoud and Rivera, 2002). Serum albumin (ALB), a plasma protein synthesized by the liver, represents an important parameter reflecting nutritional status, and reduced ALB levels in cancer patients represent a risk factor influencing tumor prognosis (Gupta and Lis, 2010). The C-reactive protein-albumin-lymphocyte (CALLY) index incorporates CRP, ALB, and lymphocyte count to reflect the inflammatory, immune, and nutritional states of patients. As a composite index, it provides a more comprehensive characterization of immune function and the balance of host inflammation, offering substantial advantages in prognostic prediction. The CALLY index has been shown to exhibit strong correlations with prognostic outcomes across diverse malignant tumors (Zhao et al., 2025; Jia et al., 2025; Yang et al., 2023; Toda et al., 2025; Jiang et al., 2024; Zhu et al., 2024; Liu et al., 2023). Owing to its rapid, straightforward, and cost-effective assessment, CALLY has been utilized for postoperative recurrence monitoring and therapeutic efficacy evaluation in various tumors, overcoming for the limitations of conventional imaging and pathological biopsy. However, evidence regarding the prognostic value of the CALLY index in EC remains limited. This investigation seeks to evaluate the predictive utility of the preoperative CALLY index in patients undergoing radical surgery for EC, thereby providing a reference for clinicians to identify high-risk patients at an early stage and develop individualized treatment strategies.

## Methods

### Study population

The training cohort of this investigation retrospectively included 288 patients who were initially diagnosed with EC and underwent radical surgery at the First Affiliated Hospital of Soochow University between January 2014 and December 2023. The validation cohort consisted of 153 EC patients who underwent radical surgery at Changzhou People’s Hospital during the same period. The inclusion criteria were as follows: (1) radical surgical treatment performed; (2) no neoadjuvant therapy administered before surgery; (3) postoperative pathological confirmation of the primary lesion as EC; and (4) completion of routine blood tests and liver and kidney function assessments within 1 week before surgery. The exclusion criteria were: (1) incomplete clinicopathological data; (2) history of infection or trauma within 2 weeks before surgery; (3) antibiotic or hormonal medication use within 2 weeks before surgery; and (4) coexistence of severe diseases, including malignancies in other organ systems. This study was executed per the Declaration of Helsinki and approved by the Medical Ethics Committee of the First Affiliated Hospital of Soochow University (Approval No.: 2025-1156). Owing to its retrospective design and complete anonymization of patient data, informed consent requirements were waived.

### Follow-up

Follow-up was executed every 3–6 months during the first 1–2 postoperative years, every 6–12 months during years 3–5, and annually thereafter. Follow-up evaluations consisted of clinical symptom assessment and imaging examinations and continued until disease recurrence, death, or the last follow-up. The cutoff date for follow-up was 31 December 2023.

### Data collection

Baseline patient characteristics (age and body mass index [BMI]), preoperative laboratory parameters (ALB, neutrophil count, lymphocyte count, monocyte count, platelet count, CRP, and serum CA125), postoperative pathological variables (histological grade, histopathological subtype, myometrial invasion, lymphatic vessel space invasion [LVSI], and International Federation of Gynecology and Obstetrics [FIGO] stage), as well as postoperative treatment information (adjuvant radiotherapy and adjuvant chemotherapy), were collected.

The calculation formulas for inflammation-related indices were as follows: CALLY index = albumin × lymphocyte/CRP; neutrophil-to-lymphocyte ratio (NLR) = neutrophil count/lymphocyte count; platelet-to-lymphocyte ratio (PLR) = platelet count/lymphocyte count; monocyte-to-lymphocyte ratio (MLR) = monocyte count/lymphocyte count; and ALB/CRP = albumin/CRP.

### Statistical methods

Statistical analyses and graphical visualizations were performed using SPSS 29.0, GraphPad 9.0, and R 4.3.0. Optimal cutoff values for CALLY, NLR, PLR, MLR, and ALB/CRP were determined by the Youden index (Van der Schouw et al., 1992). Categorical variables were presented as frequencies and percentages, and intergroup comparisons were conducted employing the chi-square test. Spearman correlation analysis was applied to evaluate associations between variables. Postoperative survival differences between high- and low-value groups were assessed using the Kaplan-Meier method, with comparative assessments conducted via the log-rank test. Multicollinearity was assessed using variance inflation factors (VIF), with a threshold of VIF <5 indicating no significant collinearity. Variables with *P* < 0.05 in the univariate Cox regression were initially included in the multivariate analysis, and then the stepwise regression method (Wald test and likelihood ratio test) was used to screen independent prognostic factors and determine the optimal fitting model. For nomogram construction, independent prognostic variables were selected using the Cox proportional hazards regression model, incorporating the Wald test and likelihood ratio test within a stepwise regression framework. Model performance was assessed using the time-dependent ROC analyses (timeROC, survival, and pROC packages in R 4.3.0). Comparisons between AUCs of different markers were conducted using the DeLong test. Internal validation was performed using calibration curves generated via 1000 Bootstrap resampling iterations. A two-sided *P*-value < 0.05 was considered statistically significant.

## Results

### Baseline characteristics of patients

The training cohort comprised 288 EC patients who underwent radical surgery, whereas the validation cohort comprised 153 EC patients. The clinicopathological characteristics were comparably distributed between the two cohorts. In the training cohort, the mean patient age was 58.1 years, with most individuals exhibiting BMI ≤25 (80.9%). Histological grade was predominantly I (47.6%), the principal histopathological subtype was endometrioid carcinoma (81.2%), and most cases were classified as FIGO stage I (68.1%) (Table 1). In the validation cohort, the mean patient age was 59.3 years, and the proportions of BMI ≤25 (84.3%), histological grade I (56.2%), endometrioid carcinoma (77.8%), and FIGO stage I (68.6%) closely resembled those observed in the training cohort (Table 1).

**TABLE 1:** Clinicopathological characteristics of patients with endometrial cancer in train cohort and validation cohort.

Characteristic	Train cohort (n = 288)		Validation cohort (n = 153)	
	No. of patients	%	No. of patients	%
BMI				
≤25	233	80.9	129	84.3
>25	55	19.1	24	15.7
Age				
≤60	151	52.7	76	49.7
>60	137	47.6	77	50.3
Histological grade				
I	137	47.6	86	56.2
II	84	29.2	31	20.3
III	67	23.3	36	23.5
Serum CA125				
≤35	187	64.9	94	61.4
>35	101	35.1	59	38.6
Histopathological subtype				
Endometrioid	77.8	234	81.2	119
Others	22.2	54	18.8	34
Myometrial invasion				
≤1/2	186	64.6	103	67.3
>1/2	102	35.4	50	32.7
LVSI				
No	194	67.4	100	65.4
Yes	94	32.6	53	34.6
FIGO stage				
I	196	68.1	105	68.6
II	37	12.8	18	11.8
III	55	19.1	30	19.6
Adjuvant radiotherapy				
No	174	60.4	102	66.7
Yes	114	38.6	51	33.3
Adjuvant chemotherapy				
No	202	70.1	110	71.9
Yes	86	29.9	43	28.1

BMI: body mass index; FIGO: international federation of gynecology and obstetrics; LVSI: lymphatic vessel space invasion.

### Link between the CALLY index and clinicopathological characteristics

In the training cohort, the preoperative cutoff value for CALLY, determined by the Youden index, was 7.5. The corresponding cutoff values were 2.9 for NLR, 154 for PLR, 0.47 for MLR, and 6.7 for ALB/CRP. Within the training cohort, CALLY exhibited statistically significant correlations with serum CA125 levels (*P* = 0.021) and LVSI (*P* = 0.011). Significant associations were also observed between CALLY and the inflammatory markers NLR, PLR, MLR, and ALB/CRP (all *P* < 0.05) (Table 2). In the validation cohort, CALLY was statistically significantly associated with the inflammatory indices NLR, PLR, MLR, and ALB/CRP (all *P* < 0.05), whereas no marked links were detected between CALLY and other clinicopathological characteristics (all *P* > 0.05) (Table 2).

**TABLE 2:** Correlations between preoperative CALLY and clinicopathological characteristics in train and validation cohort.

Clinical parameter	Train cohort				Validation cohort			
	CALLY≤7.5 (156)	CALLY>7.5 (132)	χ^2^	*P*	CALLY≤7.5 (82)	CALLY>7.5 (71)	χ^2^	*P*
BMI	​	​	0.44	0.506	​	​	0.00	0.951
≤25	124	109	​	​	69	60	​	​
>25	32	23	​	​	13	11	​	​
Age	​	​	0.00	0.961	​	​	0.06	0.812
≤60	82	69	​	​	40	36	​	​
>60	74	63	​	​	42	35	​	​
Histological grade	​	​	1.82	0.403	​	​	3.56	0.168
I	75	62	​	​	44	42	​	​
II	41	43	​	​	14	17	​	​
III	40	27	​	​	24	12	​	​
Serum CA125	​	​	5.30	0.021^*^	​	​	2.13	0.145
≤35	92	95	​	​	46	48	​	​
>35	64	37	​	​	36	23	​	​
Histopathological subtype	​	​	1.29	0.256	​	​	2.17	0.141
Endometrioid	123	111	​	​	60	59	​	​
Others	33	21	​	​	22	12	​	​
Myometrial invasion	​	​	0.31	0.578^*^	​	​	3.23	0.072
≤1/2	103	83	​	​	50	53	​	​
>1/2	53	49	​	​	32	18	​	​
LVSI	​	​	6.47	0.011^*^	​	​	0.04	0.839
No	95	99	​	​	53	47	​	​
Yes	61	33	​	​	29	24	​	​
FIGO stage	​	​	2.21	0.332	​	​	5.16	0.076
I	108	88	​	​	50	55	​	​
II	16	21	​	​	11	7	​	​
III	32	23	​	​	21	9	​	​
Adjuvant radiotherapy	​	​	3.51	0.061	​	​	0.84	0.359
No	102	72	​	​	52	50	​	​
Yes	54	60	​	​	30	21	​	​
Adjuvant chemotherapy	​	​	0.01	0.914	​	​	0.12	0.731
No	109	93	​	​	58	52	​	​
Yes	47	39	​	​	24	19	​	​
NLR	​	​	8.73	0.003^*^	​	​	13.40	<0.001^*^
NLR≤2.9	105	109	​	​	36	52	​	​
NLR>2.9	51	23	​	​	46	19	​	​
PLR	​	​	6.96	0.008^*^	​	​	12.28	<0.001^*^
PLR≤154	105	107	​	​	31	47	​	​
PLR>154	51	25	​	​	51	24	​	​
MLR	​	​	11.80	0.001^*^	​	​	7.33	0.007^*^
MLR≤0.47	123	123	​	​	24	36	​	​
MLR>0.47	33	9	​	​	58	35	​	​
Alb/CRP	​	​	30.03	<0.001^*^	​	​	26.16	<0.001^*^
Alb/CRP≤6.7	50	8	​	​	70	33	​	​
Alb/CRP >6.7	106	124	​	​	12	38	​	​

BMI: body mass index; FIGO: international federation of gynecology and obstetrics; LVSI: lymphatic vessel space invasion; NLR: neutrophil lymphocyte ratio; PLR: platelet lymphocyte ratio; MLR: monocyte lymphocyte ratio; Alb/CRP: albumin C-reactive protein radio; CALLY: C-reactive protein-albumin-lymphocyte.

### Significance of inflammatory markers on the prognosis of EC patients

**FIGURE 1:**
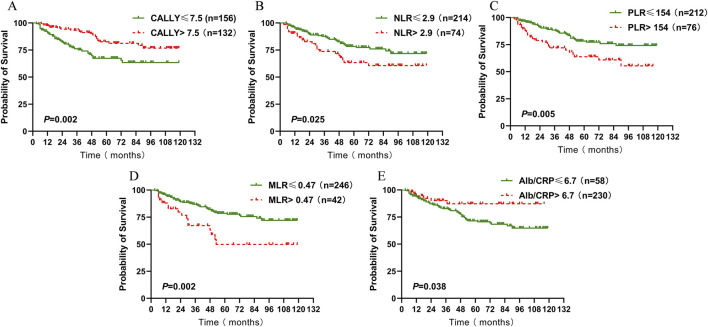
Prognostic significance of preoperative CALLY **(A)**, NLR **(B)**, PLR **(C)**, MLR **(D)**, and Alb/CRP **(E)** in patients with resectable endometrial cancer in the training cohort.

**FIGURE 2:**
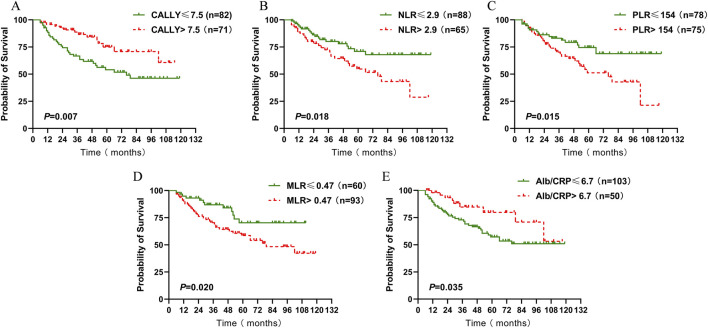
Prognostic significance of preoperative CALLY **(A)**, NLR **(B)**, PLR **(C)**, MLR **(D)**, and Alb/CRP **(E)** in patients with resectable endometrial cancer in the validation cohort.

**FIGURE 3:**
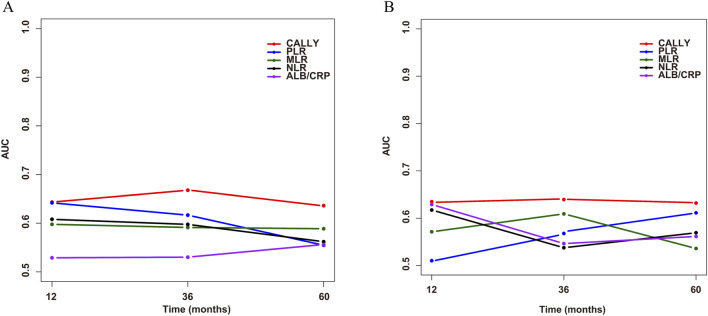
**(A)** The predictive ability of the CALLY in resectable endometrial cancer was compared with NLR, PLR, MLR and Alb/CRP by time-depend ROC in the training cohort **(B)** The predictive ability of the CALLY in resectable endometrial cancer was compared with NLR, PLR, MLR and Alb/CRP by time-depend ROC in the validation cohort.

Kaplan-Meier analyses with corresponding log-rank tests showed that in the training cohort, overall survival (OS) was markedly diminished in the CALLY ≤7.5 group versus the CALLY >7.5 group (*P* = 0.002). OS was also markedly diminished in the NLR >2.9, PLR >154, and MLR >0.47 groups versus their respective low-value groups (*P* = 0.025, *P* = 0.005, *P* = 0.002). Furthermore, the ALB/CRP ≤6.7 group demonstrated poorer survival than the >6.7 group (*P* = 0.038) (Figure 1). In the validation cohort, Kaplan–Meier survival patterns for all inflammatory markers were consistent with those of the training cohort (Figure 2). In the training cohort, univariate analyses indicated that BMI, age, histological grade, serum CA125, histopathological subtype, myometrial invasion, LVSI, FIGO stage, adjuvant chemotherapy, NLR, PLR, MLR, and ALB/CRP were markedly linked to OS. Multicollinearity diagnostics revealed that all variables had VIF values ranging from 1.15 to 2.02, well below the threshold of 5, indicating no significant multicollinearity. Subsequent multivariate Cox analysis identified BMI >25, age >60 years, grade III–II histological grade, non-endometrioid histopathological subtype, myometrial invasion >1/2, FIGO stage II, and FIGO stage III as independent risk factors for OS (Table 3). Among inflammatory markers, PLR >154, MLR >0.47, and CALLY ≤7.5 were also confirmed as independent risk factors. In the validation cohort, independent prognostic factors included BMI >25, age >60 years, grade III–II histological grade, non-endometrioid histopathological subtype, FIGO stage II, FIGO stage III, adjuvant chemotherapy, PLR >154, and CALLY ≤7.5. Notably, PLR and CALLY were the only inflammatory markers identified as independent prognostic indicators in both cohorts (Table 3). Moreover, time-dependent ROC curve analyses showed that the AUC of the CALLY index for 1-year, 3-year and 5-year survival was numerically greater than that of other inflammatory markers (NLR, PLR, MLR, ALB/CRP) in EC patients in both cohorts (Figure 3; Table 4). However, inter-marker AUC comparisons by the DeLong test showed no statistically significant differences (Table 4). These findings indicate that, relative to other systemic inflammatory indices, CALLY, integrating ALB, lymphocytes, and CRP, provides a favorable prognostic performance for postoperative survival in EC patients.

**TABLE 3:** Univariate and multivariate cox regression analyses for overall survival in patients with endometrial cancer under radical surgery.

Variables	Univariate analysis		Multivariate analysis	
	HR (95%CI)	*P* Value	HR (95%CI)	*P* Value
Train cohort				
BMI				
>25 vs. ≤25	1.80 (1.04–3.11)	0.037*	2.55 (1.39–4.69)	0.003*
Age				
>60 vs. ≤60	3.69 (2.06–6.59)	<0.001*	2.84 (1.46–5.54)	0.002*
Histological grade				
III-II vs. I	2.12 (1.65–2.72)	<0.001*	2.10 (1.55–2.86)	<0.001*
Serum CA125				
>35 vs. ≤35	2.04 (1.23–3.37)	0.006*	1.41 (0.81–2.45)	0.222
Histopathological subtype				
Others vs. Endometrioid	1.96 (1.12–3.45)	0.019*	3.52 (1.85–6.71)	<0.001*
Myometrial invasion				
>1/2 vs. ≤1/2	2.52 (1.53–4.15)	<0.001*	2.07 (1.02–4.18)	0.044*
LVSI				
Yes vs. No	1.77 (1.07–2.94)	0.028*	1.54 (0.83–2.83)	0.169
FIGO stage	​	<0.001*	​	<0.001*
II vs. I	2.24 (1.15–4.37)	0.018*	2.40 (1.08–5.32)	0.031*
III vs. I	5.31 (3.00–9.40)	<0.001*	8.31 (3.61–19.11)	<0.001*
Adjuvant radiotherapy				
Yes vs. No	1.40 (0.85–2.31)	0.184	​	​
Adjuvant chemotherapy				
Yes vs. No	2.28 (1.38–3.76)	0.001*	0.70 (0.36–1.33)	0.275
NLR				
>2.9 vs. ≤2.9	1.79 (1.07–2.99)	0.027*	0.85 (0.43–1.66)	0.630
PLR				
>154 vs. ≤154	2.05 (1.23–3.40)	0.006*	2.19 (1.22–3.93)	0.008*
MLR				
>0.47 vs. ≤0.47	2.37 (1.34–4.19)	0.003*	2.53 (1.21–5.29)	0.014*
Alb/CRP				
>6.7 vs. ≤6.7	0.42 (0.18–0.98)	0.044*	0.42 (0.18–1.01)	0.052
CALLY2				
>7.5 vs. ≤7.5	0.44 (0.26–0.75)	0.003*	0.43 (0.23–0.80)	0.008*
Validation cohort				
BMI				
>25 vs. ≤25	2.83 (1.48–5.39)	0.002*	2.44 (1.17–5.10)	0.002*
Age				
>60 vs. ≤60	3.00 (1.62–5.57)	<0.001*	2.08 (1.05–4.11)	0.037*
Histological grade				
III-II vs. I	2.14 (1.55–2.94)	<0.001*	3.24 (2.01–5.22)	<0.001*
Serum CA125				
>35 vs. ≤35	1.79 (1.01–3.17)	0.045*	0.68 (0.33–1.41)	0.304
Histopathological subtype				
Others vs. Endometrioid	3.01 (1.68–5.41)	<0.001*	4.20 (2.05–8.60)	<0.001*
Myometrial invasion				
>1/2 vs. ≤1/2	2.41 (1.36–4.30)	0.003*	1.92 (0.81–4.55)	0.141
LVSI				
Yes vs. No	1.86 (1.05–3.29)	0.033*	1.81 (0.94–3.48)	0.074
FIGO stage	​	<0.001*	​	0.002*
II vs. I	2.87 (1.38–5.96)	0.005*	4.14 (1.64–10.43)	0.003*
III vs. I	4.96 (2.55–9.67)	<0.001*	5.02 (1.84–13.69)	0.002*
Adjuvant radiotherapy				
Yes vs. No	0.87 (0.44–1.70)	0.674	​	​
Adjuvant chemotherapy				
Yes vs. No	2.18 (1.24–3.84)	0.007*	2.21 (1.12–4.37)	0.023*
NLR				
>2.9 vs. ≤2.9	1.97 (1.11–3.50)	0.021*	1.88 (0.87–4.07)	0.109
PLR				
>154 vs. ≤154	2.04 (1.13–3.68)	0.017*	2.36 (1.15–4.87)	0.020*
MLR				
>0.47 vs. ≤0.47	2.13 (1.11–4.10)	0.023*	1.74 (0.87–3.48)	0.116
Alb/CRP				
>6.7 vs. ≤6.7	0.47 (0.23–0.97)	0.040*	0.60 (0.31–1.17)	0.134
CALLY2				
>7.5 vs. ≤7.5	0.43 (0.23–0.81)	0.009*	0.35 (0.15–0.83)	0.017*

BMI: body mass index; FIGO: international federation of gynecology and obstetrics; LVSI: lymphatic vessel space invasion; NLR: neutrophil lymphocyte ratio; PLR: platelet lymphocyte ratio; MLR: monocyte lymphocyte ratio; Alb/CRP: albumin C-reactive protein radio; CALLY: C-reactive protein-albumin-lymphocyte.

**TABLE 4:** Time-dependent ROC quantitative results of inflammatory markers for 1-, 3-, and 5-year survival in training and validation cohorts.

Cohort	Times	Index	AUC (95%CI)	Index	AUC (95%CI)	DeLong *P*
Train cohort	12	CALLY	0.64 (0.54–0.74)	NLR	0.61 (0.53–0.69)	0.65
	36	​	0.67 (0.57–0.78)	​	0.60 (0.52–0.68)	0.30
	60	​	0.65 (0.55–0.76)	​	0.56 (0.46–0.66)	0.22
	12	CALLY	0.64 (0.54–0.74)	PLR	0.64 (0.52–0.77)	0.98
	36	​	0.67 (0.57–0.78)	​	0.62 (0.54–0.71)	0.47
	60	​	0.65 (0.55–0.76)	​	0.56 (0.47–0.65)	0.20
	12	CALLY	0.64 (0.54–0.74)	MLR	0.60 (0.52–0.68)	0.54
	36	​	0.67 (0.57–0.78)	​	0.59 (0.48–0.69)	0.29
	60	​	0.65 (0.55–0.76)	​	0.59 (0.52–0.65)	0.34
	12	CALLY	0.64 (0.54–0.74)	Alb/CRP	0.53 (0.45–0.61)	0.09
	36	​	0.67 (0.57–0.78)	​	0.54 (0.49–0.60)	0.03^*^
	60	​	0.65 (0.55–0.76)	​	0.56 (0.51–0.61)	0.13
Validation cohort	12	CALLY	0.63 (0.52–0.73)	NLR	0.62 (0.48–0.75)	0.91
	36	​	0.65 (0.56–0.74)	​	0.55 (0.45–0.65)	0.15
	60	​	0.63 (0.55–0.71)	​	0.57 (0.48–0.66)	0.33
	12	CALLY	0.63 (0.52–0.73)	PLR	0.51 (0.41–0.61)	0.10
	36	​	0.65 (0.56–0.74)	​	0.58 (0.48–0.68)	0.31
	60	​	0.63 (0.55–0.71)	​	0.61 (0.52–0.70)	0.74
	12	CALLY	0.63 (0.52–0.73)	MLR	0.58 (0.44–0.71)	0.57
	36	​	0.65 (0.56–0.74)	​	0.61 (0.52–0.70)	0.54
	60	​	0.63 (0.55–0.71)	​	0.54 (0.44–0.64)	0.17
	12	CALLY	0.63 (0.52–0.73)	Alb/CRP	0.63 (0.55–0.71)	0.97
	36	​	0.65 (0.56–0.74)	​	0.55 (0.44–0.66)	0.17
	60	​	0.63 (0.55–0.71)	​	0.56 (0.48–0.64)	0.23

BMI: body mass index; NLR: neutrophil lymphocyte ratio; PLR: platelet lymphocyte ratio; MLR: monocyte lymphocyte ratio; Alb/CRP: albumin C-reactive protein radio; CALLY: C-reactive protein-albumin-lymphocyte.

### Construction and evaluation of the CALLY nomogram

**FIGURE 4:**
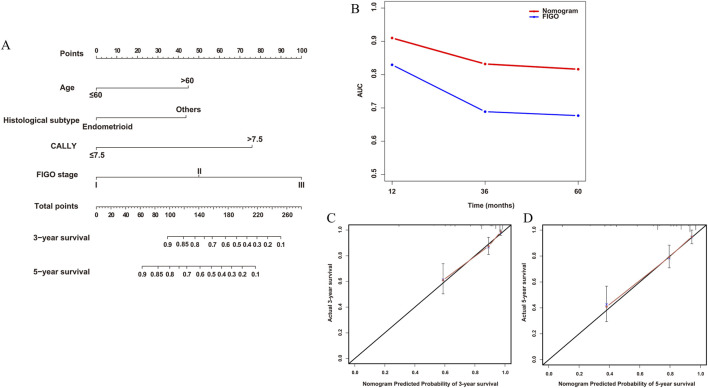
**(A)** Nomogram for overall survival (OS) in patients with resectable endometrial cancer, integrating CALLY, pathological type, age, and FIGO stage **(B)** The predictive ability of the nomogram in resectable endometrial cancer was compared with FIGO stage by time-depend ROC in the training cohort. In the training cohort, the 3-year **(C)** and 5-year **(D)** survival rates of patients with resectable endometrial cancer predicted by this nomogram were highly consistent with the actual observed values.

**FIGURE 5:**
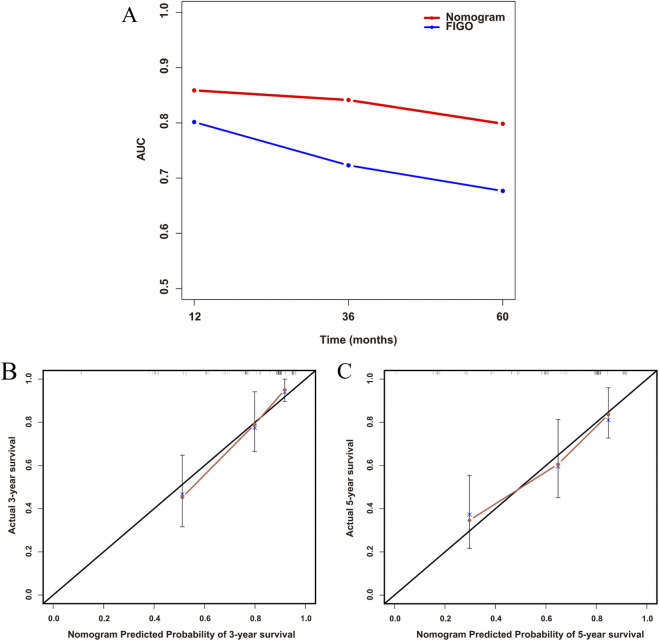
**(A)** The predictive ability of the nomogram in resectable endometrial cancer was compared with FIGO stage by time-depend ROC in the validation cohort. In the validation cohort, the 3-year **(B)** and 5-year **(C)** survival rates of patients with resectable endometrial cancer predicted by the nomogram were highly consistent with the actual observed values.

In the training cohort, Cox proportional hazards regression model was utilized to determine independent prognostic factors. The Wald test and likelihood ratio test within a stepwise regression framework were used to determine the optimal fitting model. A nomogram for predicting OS in EC patients was subsequently developed based on the CALLY index, histopathological subtype, age, and FIGO stage (Figure 4A). In the training cohort, the AUC values of the nomogram for predicting 1-year, 3-year and 5-year survival were 0.91, 0.84 and 0.81, respectively, both exceeding the corresponding AUC values for FIGO staging (0.80, 0.69, and 0.68) (Figure 4B; Table 5). DeLong test confirmed that the AUC of the nomogram was significantly higher than that of FIGO staging alone at all time points (*P* < 0.05), indicating better prognostic discrimination (Table 5). Calibration curves indicated strong agreement between nomogram-predicted and observed 3-year and 5-year survival outcomes (Figures 4C,D). In the validation cohort, the AUC values for predicting 1-year, 3-year and 5-year survival were 0.85, 0.82 and 0.78, respectively, also surpassing the AUC values associated with FIGO staging (0.78, 0.72 and 0.69) (Figure 5A; Table 5). Calibration curves in the validation cohort further demonstrated close alignment between predicted probabilities and actual outcomes (Figures 5B,C). Collectively, these observations indicate that the nomogram provides a reliable and effective tool for predicting postoperative OS in EC patients.

**TABLE 5:** Time-dependent ROC quantitative results of nomogram and FIGO stage for 1-, 3-, and 5-year survival in training and validation cohorts.

Cohort	Times	Index	AUC (95%CI)	Index	AUC (95%CI)	DeLong *P*
Train cohort	12	Nomogram	0.91 (0.85–0.97)	FIGO	0.80 (0.70–0.91)	0.03^*^
	36	​	0.84 (0.70–0.91)	​	0.69 (0.61–0.78)	<0.01^*^
	60	​	0.81 (0.75–0.87)	​	0.68 (0.61–0.76)	<0.01^*^
Validation cohort	12	Nomogram	0.85 (0.75–0.95)	FIGO	0.78 (0.63–0.92)	0.32
	36	​	0.82 (0.74–0.90)	​	0.72 (0.62–0.81)	0.03^*^
	60	​	0.78 (0.70–0.86)	​	0.69 (0.60–0.78)	0.03^*^

FIGO: international federation of gynecology and obstetrics.

## Discussion

EC is a highly heterogeneous malignant tumor. Although FIGO staging, histological grading, and molecular classification are widely applied in clinical practice to guide treatment and estimate prognosis, these indicators remain limited in predictive accuracy. Previous studies have demonstrated that systemic inflammatory markers are closely associated with the prognosis of EC (Cummings et al., 2015; Ahn et al., 2022; Cong et al., 2020; Nishio et al., 2024; Choi et al., 2025). Cummings et al. reported that NLR and PLR were independent prognostic indicators for EC, and that their combined application provided additional value for patient risk stratification (Cummings et al., 2015). Ahn et al. further showed that elevated MLR was markedly linked to poorer clinical outcomes in stage I EC patients and served as an independent prognostic biomarker in this population (Ahn et al., 2022). Additionally, nomograms integrating NLR, PLR, MLR, and other clinicopathological characteristics have been reported to improve the prediction of OS in EC patients (Cong et al., 2020). Compared with previous research, the present study systematically evaluated and externally validated the prognostic significance of the CALLY index in EC. A study design incorporating a training cohort (n = 288) and an external validation cohort (n = 153) was adopted, enhancing the reliability and generalizability of the findings. The three parameters constituting the CALLY index (CRP, albumin, and lymphocytes) are routinely assessed preoperatively, easily obtainable, cost-efficient, and feasible for clinical application. A nomogram with strong predictive performance was subsequently established and validated, demonstrating markedly superior prognostic discrimination compared with traditional FIGO staging.

In recent years, the CALLY index, an indicator reflecting host systemic inflammation, immune competence, and nutritional status, has exhibited substantial potential for tumor prognosis prediction. By integrating three core parameters: CRP, lymphocyte count, and ALB, the CALLY index provides a comprehensive quantification of the “inflammation–immunity–nutrition axis”, a conceptual framework that aligns closely with contemporary understanding of regulatory mechanisms within the tumor microenvironment. CRP can facilitate malignant tumor progression through activation of multiple signaling pathways (including the FcγRs/MAPK/ERK, FcγRs/IL-6/AKT/STAT3, and FcγRs/NF-κB/NLRP3 pathways) (Socha et al., 2021), and evidence has demonstrated that both CRP levels and dynamic changes serve as independent prognostic factors for EC (Socha et al., 2021). Lymphocytes play a pivotal role in cell-mediated immunity, mediating cytotoxic activity and cytokine secretion that suppress tumor proliferation, metastasis, and growth (Mantovani et al., 2008). ALB, synthesized by the liver, is a widely used marker of nutritional status and functions as an important antioxidant, detoxifying molecule, and transporter of essential metabolites. Owing to its long half-life, it is frequently applied to assess nutritional reserve and disease burden. Studies have indicated that hypoalbuminemia in cancer patients arises from cytokine-driven suppression of albumin synthesis and enhanced catabolism, partially reflecting systemic inflammatory status, with albumin levels correlating with tumor prognosis (Haskins et al., 2017). The combined incorporation of these three components enables the CALLY index to overcome the limitations inherent to single-parameter assessments, thereby capturing a more integrated representation of host–tumor biological interactions. The prognostic superiority of the CALLY index over other systemic inflammatory markers has been demonstrated across malignant tumors (Jia et al., 2025; Yang et al., 2023; Toda et al., 2025; Jiang et al., 2024; Zhu et al., 2024; Liu et al., 2023; Iida et al., 2022). In hepatocellular carcinoma, the CALLY index is a prognostic factor for postoperative patients and offers greater predictive value than ALB/CRP, NLR, PLR, MLR, and LCR (Iida et al., 2022). In esophageal cancer, the CALLY index is independently and positively associated with OS, and nomograms incorporating it outperform TMN staging in predicting OS (Jia et al., 2025). In colorectal cancer, the CALLY index is independently associated with OS, with prognostic capability exceeding that of mGPS, NLR, SII, and PLR, and nomogram models provide more accurate prognostic estimation than TNM staging (Yang et al., 2023). The CALLY index also functions as an independent prognostic indicator for OS and RFS in postoperative gastric cancer (Toda et al., 2025). Notably, the prognostic significance of the CALLY index in EC may be partly due to its capacity to sensitively reflect subclinical cancer cachexia, a critical pathophysiological process in patients with advanced or recurrent EC. Cancer cachexia is characterized by progressive metabolic exhaustion, systemic inflammation, and severe muscle wasting, in which the interplay of the inflammatory, metabolic, and nutritional axis serves as a core driving mechanism (doi:10.1002/mef2.70008). Hypoalbuminemia, a central component of the CALLY index, not only indicates exacerbated systemic inflammation but also represents a hallmark of nutritional depletion and muscle atrophy in cancer cachexia. Meanwhile, elevated CRP and decreased lymphocyte counts further reflect the systemic inflammatory–immune disturbance that accelerates cachectic progression. As a composite indicator incorporating albumin, CRP, and lymphocytes, the CALLY index effectively captures the integrated changes of inflammation, immunity, and nutrition in EC patients, thereby enabling the early identification of subclinical cachexia that precedes overt phenotypic manifestations. This may partially explain the favorable prognostic performance of the CALLY index in predicting survival outcomes in patients with EC (Gongchang Zhang et al., 2024). This study systematically confirmed, within a multicenter retrospective cohort, that the prognostic performance of the CALLY index following radical EC surgery surpasses that of traditional systemic inflammatory indicators (NLR, PLR, MLR, and ALB/CRP), consistent with evidence reported in other tumors. To further improve individualized risk estimation, a nomogram integrating the CALLY index, histopathological subtype, age, and FIGO staging was developed for predicting OS in EC. This model demonstrated strong discriminative ability and calibration in both the training and validation cohorts. Its AUC values markedly exceeded those of FIGO staging alone. Calibration analyses further verified excellent concordance between predicted and observed survival outcomes. These findings suggest that the proposed nomogram can more precisely quantify patient-specific prognostic risk and provide a robust evidence-based framework and practical tool to support precision clinical management of EC.

Despite these findings, several limitations remain in this study: 1) as a retrospective analysis, inherent selection bias and unavoidable incompleteness in data recording may have occurred; 2) the cutoff value of CALLY = 7.5 was established using the Youden index in the training cohort, which is a data-driven method and may carry potential risks of overfitting and limited generalizability. Although this cutoff was validated in an external cohort, its universal applicability still requires confirmation in larger and more diverse populations; 3) because the study population originated from only two medical centers, larger multicenter prospective investigations are needed to validate its applicability across varied populations; and 4) this study did not incorporate EC molecular subtype information such as POLE mutations and dMMR, which have become important determinants in contemporary EC prognostic evaluation. As the molecular classification has been widely validated to independently predict survival, recurrence risk, and treatment response in EC patients, the absence of these molecular indicators may reduce the comprehensive prognostic stratification ability of our nomogram. The predictive accuracy and clinical applicability of the current model could be further improved by integrating molecular subtypes in future research. Future research integrating the CALLY index with molecular subtype characteristics may enable the development of more robust prognostic models; and 5) the sample size of the external validation cohort (n = 153) is relatively small, which may restrict the statistical power and generalizability of the findings. Although the baseline clinicopathological characteristics of the validation cohort were well balanced with those of the training cohort, a larger-scale external validation cohort is still needed to further consolidate the reliability of our results.

This multicenter cohort study demonstrated that the preoperative CALLY index functions as an independent prognostic risk factor for OS in patients undergoing radical surgery for EC, exhibiting superior predictive capability compared with traditional systemic inflammatory markers. The nomogram developed on the basis of the CALLY index substantially improved the prognostic stratification accuracy of conventional FIGO staging. Owing to its accessibility, simplicity, and low cost, this index may serve as a novel biomarker for preoperative prognostic evaluation in EC. The established nomogram may support clinicians in determining individualized therapeutic and follow-up strategies, thereby providing a practical tool for precision diagnosis and management of EC.

## Data Availability

The raw data supporting the conclusions of this article will be made available by the authors, without undue reservation.

## References

[B1] Ahn J. H. Lee S. J. Yoon J. H. Park D. C. Kim S. I. (2022). Prognostic value of pretreatment systemic inflammatory markers in patients with stage I endometrial cancer. Int. J. Med. Sci. 19 (14), 1989–1994. 10.7150/ijms.78182 36483600 PMC9724239

[B2] Balkwill F. Mantovani A. (2001). Inflammation and cancer: back to virchow? Lancet 357 (9255), 539–545. 10.1016/S0140-6736(00)04046-0 11229684

[B3] Bray F. Laversanne M. Sung H. Ferlay J. Siegel R. L. Soerjomataram I. (2024). Global cancer statistics 2022: GLOBOCAN estimates of incidence and mortality worldwide for 36 cancers in 185 countries. CA Cancer J. Clin. 74 (3), 229–263. 10.3322/caac.21834 38572751

[B4] Choi M. Lee S. W. Park W. Lee Y. S. Lee S. H. Lee J. H. (2025). Can posttreatment blood inflammatory markers predict poor survival in gynecologic cancer? a systematic review and meta-analysis. Front. Immunol. 16, 1676838. 10.3389/fimmu.2025.1676838 41194932 PMC12583213

[B5] Cong R. Kong F. Ma J. Li Q. Wu Q. Ma X. (2020). Combination of preoperative neutrophil-lymphocyte ratio, platelet-lymphocyte ratio and monocyte-lymphocyte ratio: a superior prognostic factor of endometrial cancer. BMC Cancer 20 (1), 464. 10.1186/s12885-020-06953-8 32448185 PMC7245911

[B6] Crosbie E. J. Kitson S. J. McAlpine J. N. Mukhopadhyay A. Powell M. E. Singh N. (2022). Endometrial cancer. Lancet 399 (10333), 1412–1428. 10.1016/S0140-6736(22)00323-3 35397864

[B7] Cummings M. Merone L. Keeble C. Burland L. Grzelinski M. Sutton K. (2015). Preoperative neutrophil:lymphocyte and platelet:lymphocyte ratios predict endometrial cancer survival. Br. J. Cancer 113 (2), 311–320. 10.1038/bjc.2015.200 26079303 PMC4506386

[B8] Gongchang Zhang F. H. Huang T. Ma X. Cheng Y. Liu X. Jiang W. (2024). The recent development, application, and future prospects of muscle atrophy animal models. MedComm – Future Med. 3 (4), e70008. 10.1002/mef2.70008

[B9] Gupta D. Lis C. G. (2010). Pretreatment serum albumin as a predictor of cancer survival: a systematic review of the epidemiological literature. Nutr. J. 9, 69. 10.1186/1475-2891-9-69 21176210 PMC3019132

[B10] Haskins I. N. Baginsky M. Amdur R. L. Agarwal S. (2017). Preoperative hypoalbuminemia is associated with worse outcomes in colon cancer patients. Clin. Nutr. 36 (5), 1333–1338. 10.1016/j.clnu.2016.08.023 27612919

[B11] Iida H. Tani M. Komeda K. Nomi T. Matsushima H. Tanaka S. (2022). Superiority of CRP-albumin-lymphocyte index (CALLY index) as a non-invasive prognostic biomarker after hepatectomy for hepatocellular carcinoma. HPB Oxf. 24 (1), 101–115. 10.1016/j.hpb.2021.06.414 34244053

[B12] Jia P. Shen F. Zhao Q. Wu X. Sun K. Wang X. (2025). Association between C-reactive protein-albumin-lymphocyte index and overall survival in patients with esophageal cancer. Clin. Nutr. 45, 212–222. 10.1016/j.clnu.2024.12.032 39837076

[B13] Jiang T. Sun H. Xu T. Xue S. Xia W. Xiao X. (2024). Significance of pre-treatment CALLY score combined with EBV-DNA levels for prognostication in non-metastatic nasopharyngeal cancer patients: a clinical perspective. J. Inflamm. Res. 17, 3353–3369. 10.2147/JIR.S460109 38803689 PMC11129745

[B14] Liu X. Y. Zhang X. Zhang Q. Ruan G. T. Liu T. Xie H. L. (2023). The value of CRP-albumin-lymphocyte index (CALLY index) as a prognostic biomarker in patients with non-small cell lung cancer. Support Care Cancer 31 (9), 533. 10.1007/s00520-023-07997-9 37610445

[B15] Mahmoud F. A. Rivera N. I. (2002). The role of C-reactive protein as a prognostic indicator in advanced cancer. Curr. Oncol. Rep. 4 (3), 250–255. 10.1007/s11912-002-0023-1 11937016

[B16] Mantovani A. Allavena P. Sica A. Balkwill F. (2008). Cancer-related inflammation. Nature 454 (7203), 436–444. 10.1038/nature07205 18650914

[B17] Nishio S. Murotani K. Yamagami W. Suzuki S. Nakai H. Kato K. (2024). Pretreatment systemic inflammatory markers predict survival in endometrial cancer: a Japanese gynecologic oncology group 2043 exploratory data analysis. Gynecol. Oncol. 181, 46–53. 10.1016/j.ygyno.2023.12.007 38113633

[B18] Socha M. W. Malinowski B. Puk O. Wartega M. Bernard P. Nowaczyk M. (2021). C-reactive protein as a diagnostic and prognostic factor of endometrial cancer. Crit. Rev. Oncol. Hematol. 164, 103419. 10.1016/j.critrevonc.2021.103419 34245857

[B19] Toda M. Musha H. Suzuki T. Nomura T. Motoi F. (2025). Impact of C-reactive protein-albumin-lymphocyte index as a prognostic marker for the patients with undergoing gastric cancer surgery. Front. Nutr. 12, 1556062. 10.3389/fnut.2025.1556062 40144575 PMC11937851

[B20] Van der Schouw Y. T. Verbeek A. L. Ruijs J. H. (1992). ROC curves for the initial assessment of new diagnostic tests. Fam. Pract. 9 (4), 506–511. 10.1093/fampra/9.4.506 1490547

[B21] Yang M. Lin S. Q. Liu X. Y. Tang M. Hu C. L. Wang Z. W. (2023). Association between C-reactive protein-albumin-lymphocyte (CALLY) index and overall survival in patients with colorectal cancer: from the investigation on nutrition status and clinical outcome of common cancers study. Front. Immunol. 14, 1131496. 10.3389/fimmu.2023.1131496 37063910 PMC10098202

[B22] Zhao H. Yin B. Li X. R. Liu X. Y. Bu Z. T. Shi H. P. (2025). The CRP-albumin-lymphocyte index provides enhanced prognostic value in liver cancer compared to the TNM staging system. Sci. Rep. 15 (1), 20090. 10.1038/s41598-025-03985-7 40537551 PMC12179261

[B23] Zhu D. Lin Y. D. Yao Y. Z. Qi X. J. Qian K. Lin L. Z. (2024). Negative association of C-reactive protein-albumin-lymphocyte index (CALLY index) with all-cause and cause-specific mortality in patients with cancer: results from NHANES 1999-2018. BMC Cancer 24 (1), 1499. 10.1186/s12885-024-13261-y 39639229 PMC11619214

